# Wrist-based wearable oscillometric blood pressure monitoring during Ramadan: a comparative study with standard devices

**DOI:** 10.3389/fcvm.2026.1655897

**Published:** 2026-04-22

**Authors:** Eyad Talal Attar

**Affiliations:** Department of Electrical and Computer Engineering, King Abdulaziz University, Jeddah, Saudi Arabia

**Keywords:** blood pressure, cardiovascular health, circadian variation, diastolic, Huawei watch d, non-invasive measurement, physiological monitoring, Ramadan fasting

## Abstract

The determination of blood pressure (BP) is crucial for the regulation of cardiovascular health status, particularly under the condition of physiological stress as seen during Ramadan fasting. Conventional BP measurement techniques are, accurate, intermittent and do not reflect the dynamic changes of fasting and rehydration. Wrist—based wearable oscillometric blood pressure monitoring devices, incorporating miniaturized inflatable cuffs, provide a portable and non—invasive approach for assessing BP outside traditional clinical settings. To assess the reliability of a consumer—grade wearable device (Huawei Watch D) for measuring systolic and diastolic blood pressure during Ramadan fasting, and to compare its performance with a validated electronic upper-arm monitor after pre-fast and post—fast conditions (Sahoor and If tar).

**Methods:**

A within subject, repeated—measures investigation was performed with 101 healthy fasting subjects (ages 22—70). Each participant recorded BP twice daily during Sahoor and If tar using both the Huawei Watch D and an upper—arm electronic monitor, both are FDA-approved. Statistical methods comprised paired t tests, as well as Pearson correlations and Bland—Altman plots. To evaluate prediction accuracy and discover subgroups, we also used machine learning approaches (linear regression and K—means clustering).

**Results:**

Compared with the electronic upper-arm monitor, the Huawei Watch D demonstrated minimal mean bias for both systolic and diastolic blood pressure (SBP: +0.9 to +1.4 mmHg; DBP: −0.6 to −0.2 mmHg). However, SBP exhibited wide limits of agreement (±18–21 mmHg), whereas DBP showed narrower dispersion (±11–13 mmHg). Machine-learning models achieved moderate prediction accuracy for DBP (MAE ≈ 4.3–6.1 mmHg, R² up to 0.74), while SBP prediction remained less accurate (MAE ≈ 6.5–8.9 mmHg, R² up to 0.64).However, SBP exhibited wide limits of agreement (±20 mmHg), limiting diagnostic interchangeability. DBP showed narrower error dispersion, supporting the use of wearable devices for trend monitoring and screening rather than clinical diagnosis.

**Conclusions:**

Smartwatch BP estimates are associated with standard devices, however, individual differences are considerable and therefore do not allow for diagnostic interchangeability. Wearables, such as the Huawei Watch D, provide the interesting data on the BP trend during fasting and rehydration, especially in diastolic pressure. These devices may assist with culturally appropriate monitoring and early identification of BP abnormalities however are in need of validation in a clinical context.

## 1 Introduction

The force of blood flow through arteries and veins, which physicians frequently check as a diagnostic measurement because it correlates well with the force of the heartbeat and the size of blood vessels. Sphygmomanometry is the widely used method for blood pressure measurement using the upper arm as a measuring site, focusing either on core-based auscultatory methods or on oscillometric approaches. Used widely in clinical and at—home settings, both test methods are known as the gold standard due to their established accuracy (1). However, traditional techniques for BP measurements are intermittent and user—dependent, which may not be applicable in some situations and can be inconvenient, uncomfortable, and even difficult for certain individuals, such as the elderly, obese, or those with physical or cognitive handicaps (2). During the 9th month of the Islamic Hijri calendar, Muslims worldwide fast by abstaining from food and liquids (including oral medications) daily from dawn (Suhoor) to sunset (If tar). This disruption of daily living results in a range of dramatic physiological and behavioural alterations including changes in the sleep—wake cycle, feeding, hydration state and medication adherence. The combined effects of circadian rhythm shifts together with sympathetic nerve activity alterations and plasma volume fluctuations, create complex disturbances in cardiovascular dynamics that impact blood pressure regulation (3).

More recent investigations into the cardiovascular effects of Ramadan fasting have reported heterogeneous blood pressure responses, influenced by age, hydration, sleep disruption, and baseline cardiovascular status. Post —2020 studies have demonstrated that while some individuals exhibit stable or reduced BP during Ramadan, others particularly those with undiagnosed or borderline hypertension—experience elevated systolic or diastolic values during fasting or rehydration phases (4, 5).

There are conflicting results for studies on the impact of Ramadan fasting on the BP. Others report decreases in SBP and DBP, associated with lower weight, lower caloric intake, and improved metabolic profiles such as lipid profile and insulin sensitivity (6, 7). Others have reported increased BP or increased BP variability possibly related with dehydration, sleep deprivation, or less antihypertensive drug adherence during fasting hours (8, 9). These inconsistencies may be due to variations in study method, sample size, climate of local area, dietary habits, and time of measurements.

Importantly, in the majority of studies previously published measurements of clinic or home BP were only measured at single (and often unmentioned) timepoints. So that such studies have not addressed these fluctuating BP patterns which can vary greatly over periods of fasting and feeding. In addition, monitoring itself can affect the BP (so-called “white coat effect) and measuring the BP with cuffs maybe challenging from a cultural or logistical perspective on a fixed schedule during the daytime in Ramadan, as (before iftar when people are tired and thirsty).

To address these challenges, continuous ambulatory BP monitoring technologies have emerged as a practiceable substitute. Wearable devices including smartwatches and fitness bands use optical sensors (photoplethysmography, PPG) along with machine learning models for non-invasive continuous BP estimation (10, 11). Although existing wearables are not quite as accurate as upper-arm oscillometric devices and are largely not approved for diagnostic purposes. They offer useful psychometric estimates of BP trends during free—living activity. They are especially useful during Ramadan, when traditional methods of measurement could be impossible. If proven valid, wearable BP devices have the potential to facilitate more detailed evaluations cardiovascular health during Ramadan, which could benefit both clinical studies and health monitoring on an individual level. For example, hypertensive individuals could be remotely followed for BP changes and drug intake timing, and researchers may further characterize fasting—associated cardiovascular responses with high time resolution. These may eventually lead to better management of chronic diseases in the Muslim population during Ramadan and be of value in the generation of culturally acceptable health advice.

In recent years, the rapid expansion of digital health technologies and wearable blood pressure monitoring systems has accelerated their integration into both clinical research and community health applications.

Recent studies (2021—2024) have emphasized the growing role of wrist—based wearable devices for longitudinal BP trend monitoring, early risk screening, and out—of—clinic assessment, while also highlighting persistent challenges related to measurement accuracy, inter—individual variability, and regulatory validation (12, 13).

Recent advances in wearable BP monitoring have led to the development of wrist—based devices employing oscillometric measurement principles through miniaturized inflatable cuffs. Unlike truly cuff less approaches that rely solely on surrogate physiological signals (14). These devices extend the well—established oscillometric methodology into a compact, wearable form factor. This approach allows for repeated, out—of—clinic BP measurements while maintaining closer methodological alignment with clinically validated upper-arm electronic sphygmomanometers. Although the Huawei Watch D is a wearable device, it is not classified as cuff less, as BP estimation is performed using an integrated inflatable cuff based on oscillometric principles. Only a small number have also assessed their reliability between fasting and rehydration during Ramadan. Finally, although some studies analyzed the difference between fasting and nonfasting BP profiles, none (to the best of our knowledge) performed a within—subject comparison based on both smartwatch and reference systems at specific times (Sahoor and If tar) of the day during Ramadan. Additionally, these comparisons in most prior studies have not involved close— time—point measurements from the pre—fast state to the post fast state to characterize the physiologic change as it is occurring. Such a discrepancy is particularly important, given the increasing use of wearable health data for both clinical and personal decisions. To enable acute health management as well as to promote long—term disease preventive strategies, it's indispensable that these instruments are reliable in different physiological conditions. In addition, the application of body-worn BP monitoring data to identify physiological patterns, to cluster subjects according to risk, or to predict established clinical measurements by explorative machine learning approaches has not been thoroughly investigated in the context of Ramadan fasting. Addressing this shortfall can improve the integration of wearable health tech in culturally diverse populations and fasting—related health (15).

This study aims to fill the aforementioned gaps. First, evaluating the reliability of smartwatch—derived BP readings during Ramadan, specifically comparing fasting (Sahoor) and rehydration (Iftar) periods within the same individuals. Second, this study compared smartwatch estimates with those from a validated electronic upper—arm device to assess agreement, bias, and correlation using statistical methods, including paired *t*-tests, Pearson correlation coefficients, and Bland—Altman analysis. Finally, applying supervised machine learning (regression) to predict electronic BP values from wearable data, and unsupervised clustering (K means) to identify physiological subgroups among fasting individuals.

By combining inferential statistics and data-driven learning approaches, this study seeks to assess the feasibility of using consumer—grade wearable devices for BP monitoring during fasting and to support their potential role in personalized, culturally appropriate health tracking across diverse populations.

Based on prior evidence regarding fasting—related cardiovascular variability and the known limitations of wearable BP monitoring, the present study was designed to test the following hypotheses:

HI: Diastolic BP exhibits greater variability across fasting states (Sahoor vs. Iftar) than systolic blood pressure.

H2: Measurement error between wrist—based wearable and upper arm electronic BP measurements differs across age groups and baseline BP categories.

H 3: Non-linear machine learning models outperform linear regression in predicting reference BP values from wearable—derived measurements.

## 2 Method

### 2.1 Study design

This study employed a within—subject, repeated—measures design to assess the reliability of wearable BP monitors during Ramadan, comparing readings during the fasting (Sahoor) and rehydration (Iftar) periods. Each participant was evaluated under two distinct physiological states—pre-fast and post-fast allowing for intra-individual comparisons across time and device type. BP measurements were collected using both a clinically validated electronic upper—arm monitor and a commercially available smartwatch, enabling assessment of device consistency and the effects of fasting.

### 2.2 Participants

In all, 101 supjects were sampled from the community and university through convenience sampling. The population consisted of 78 men and 23 women, and their ages varied from 22 to 70 years (mean 33. 9 ± 13. 2 years). Forty-seven percent of participants rated their health as excellent (47) or very good (38). Educational level was diverse: 35 participants had completed a bachelor' s degree; 18 had high school graduates diploma and 10 did not complete high school. Thirteen were students, and 7 were unemployed; marital status was virtually equally divided (53 married, 44 single).

The research project received ethical approval from King Abdulaziz University' s Institutional Review Board (IRB# 02 01—05—24) and all study participants provided written informed consent following the Declaration of Helsinki principles. Participant demographic characteristics, including age, sex, weight, height, and body mass index, are summarized in Table 1. Also, the Table 2 shows participant characteristics by age group.

**Table 1:** Participant demographics.

Number of participants (*n*)	101
Age (years)	34.8 ± 9.6
Sex	Male: 58 (57.4%)/female: 43 (42.6%)
Weight (kg)	76.2 ± 14.1
Height (cm)	168.5 ± 8.9
Body mass index (kg/m²)	26.8 ± 4.7

Continuous variables are reported as mean ± standard deviation (SD). BMI was calculated as weight (kg) divided by height squared (m²).

**Table 2:** Participant characteristics stratified by age group.

Variable	Overall	≤30 yrs	31–45 yrs	>45 yrs
*N*	101	34	41	26
BMI (kg/m²)	26.8 ± 4.7	25.1 ± 3.9	27.0 ± 4.4	28.9 ± 5.2

Values are reported as mean ± standard deviation (SD). Age groups were selected to reflect young, middle-aged, and older adult categories commonly used in cardiovascular research.

### 2.3 Blood pressure measurement protocol

BP was recorded twice daily—before fasting (Sahoor) and after breaking fast (Iftar). Sahoor readings were collected on the point of time before the crack of dawn, and Iftar measurements were recorded after nearly 10 min of having a standard meal consisting of 10 grams of dates and 300 mL of water. To minimize physiologic fluctuations, subjects were seated and rested for at least five minutes before each set of measurements.

In every session, two pairs of systolic and diastolic BP measurements were obtained using two instruments; a validated electronic upper-arm monitor and the Huawei Watch D smartwatch (16). The electronic monitor was used first, followed by the smartwatch, which was worn on the non-dominant wrist to reduce muscular and mechanical interference.

#### 2.3.1 Huawei watch D—blood pressure measurement principle

The Huawei Watch D measures BP using a wrist-based oscillometric method implemented through a miniaturized inflatable cuff embedded within the wrist strap, operating on the same fundamental principle as conventional upper-arm electronic sphygmomanometers. During each measurement, an integrated micro air pump inflates the wrist cuff to apply controlled external pressure over the radial artery. Arterial pressure oscillations generated during cuff deflation are detected and analyzed by the device's internal pressure sensors to estimate systolic and diastolic BP. The device also incorporates PPG sensors for auxiliary cardiovascular monitoring, such as heart rate and signal quality assessment. This cuff-based oscillometric configuration differentiates the Huawei Watch D from Wrist-Based wearable oscillometric BP devices and enables closer methodological alignment with clinically validated upper-arm oscillometric monitors (17). The Huawei Watch D has been evaluated in independent validation studies that follow measurement accuracy requirements aligned with ISO 81060-2 protocols for non-invasive BP monitoring. However, at the time of this study, the device has not been formally certified under the Association for the Advancement of Medical Instrumentation (AAMI) or the British Hypertension Society (BHS) grading systems for diagnostic BP measurement. Consequently, the Huawei Watch D should be regarded as a research-use and general wellness device, rather than a clinical-grade diagnostic instrument.

#### 2.3.2 Electronic blood pressure monitor

The electronic BP monitor employed in this study is a clinically validated and FDA-approved upper-arm device that utilizes the oscillometric technique. This equipment has High level of accuracy and repeatability and it is approved by the American Heart Association especially in clinical and research conditions (9). It has an LCD for systolic and diastolic readings and has a broad measurement range of 30–280 mmHg for systolic pressure and 20–180 mmHg for diastolic pressure. The accuracy of the device is ±3 mmHg.

Consensus clinical guidelines require healthcare providers to position the cuff on the upper arm at the level of the heart during both physical body and simulator based activities, to minimize positional bias and to guarantee that the reading can be repeated.

### 2.4 Data collection and processing

BP measurements took place indoors within quiet controlled conditions which minimized environmental variability while maintaining a constant temperature of 22 °C. To maintain data reliability participants were instructed to remain silent without external disturbances during measurement procedures. Every individual who participated provided their agreement to join the research study.

Responses were collected via standardized Google Forms and exported to Microsoft Excel for pre-processing and analysis. For each participant, eight BP measurements were obtained, four for systolic and four for diastolic, two from the automatic upper-arm monitor and two from the Huawei Watch D smartwatch, from two phases of the day—Sahoor (before fasting) and Iftar (after fasting) periods. Demographic and self-reported health information, including age, sex, marital status, education, and subjective health were obtained as well as BP readings. Unique anonymized email IDs were assigned to participants to ensure data confidentiality and link participants' responses across sessions.

Quality checks were performed to clean up the data so that the data are correct and usable. Entries that contained values that were previously omitted (e.g., systolic BP <80 mmHg and diastolic BP <40 mmHg) were removed from subsequent analysis. The remaining valid readings were normalized and organized for statistical processing.

### 2.5 Statistical analysis

The statistical processing was implemented in Python 3.11, using several libraries panda, scipy. stats, statsmodels, seaborn, and matplotlib. Preprocessing of the data involved removal of physiologically implausible data points (e.g., values outside of 1 standard deviation over 1TU of underlying values) and averaging of measurements were taken for each individual per device (and visit). Descriptive statistics (mean and standard-deviation) for SBP and DBP across the different devices and time points were computed to provide an overview in terms of baseline variability, as well as to assess potential session-specific drift. Paired sample *t*-tests were used to examine the differences between Sahoor and Iftar readings within participants and differences between smartwatch and electronic device readings at each timepoint.

Separate tests were conducted for systolic and diastolic values. A significance threshold of *α* = 0.05 was used. The paired *t*-test is appropriate for within-subject comparisons and assumes normally distributed differences.

Pearson correlation coefficients were computed to assess the linear relationship between smartwatch and electronic device readings for systolic and diastolic measures across timepoints. Correlation values above 0.70 were interpreted as strong, though correlation alone does not indicate clinical agreement.

Bland–Altman plots were constructed to evaluate the agreement between devices. The mean difference (bias) and the 95% limits of agreement (mean ± 1.96 × SD) were calculated for each pair of measurements. This work contributes to the understanding of systematic bias and measurement variance, of special importance in clinical validation.

Boxplots were presented for distributional comparison, scatter plots with regression lines were depicted to explore relationships and Bland–Altman plots for visual inspection of device agreement. All plots followed standardized color schemes to enhance clarity.

### 2.6 Machine learning methods

To explore the predictive capability of wearable BP measurements and to identify latent physiological patterns, supervised and unsupervised machine learning approaches were applied. Linear regression models were constructed to predict reference BP values obtained from the electronic upper-arm monitor.

To ensure robust model evaluation and prevent data leakage, the dataset was split at the participant level into training (70%) and testing (30%) subsets using stratified random sampling. This approach ensured that measurements from the same individual were not simultaneously present in both training and testing sets.

Model performance was evaluated on the held-out test set using mean squared error (MSE) and the coefficient of determination (R²). These metrics were selected to quantify both absolute prediction error and the proportion of variance explained by the wearable-derived inputs.

In addition to linear regression, non-linear supervised machine learning models were implemented to capture potential non-linear relationships between wearable-derived measurements and reference BP values. These included Support Vector Regression (SVR) with a radial basis function (RBF) kernel, Gradient Boosting Regression, and Elastic Net regression, which combines L1 and L2 regularization to balance feature selection and model stability.

Input features for all models consisted of smartwatch-derived systolic and DBP values, along with measurement timing (Sahoor or Iftar) and participant age group where applicable. The output targets were the corresponding systolic and DBP values obtained from the electronic upper-arm monitor.

Model performance was evaluated using k-fold cross-validation (k = 5) at the participant level to avoid data leakage. Evaluation metrics included MAE, RMSE, and R². Hyperparameters were optimized using grid search within the training folds.

## 3 Result

### 3.1 Descriptive statistics

The mean and standard deviation for the systolic and diastolic BP measurements by devices and visits are presented in Table 3. Systolic readings were higher for the second measurement round than for the initial round; to a small extent, correlated differences were observed for both electronic and watch devices on average (mean difference = 3.5 mm Hg and 3.3 mm Hg, respectively) vs. the first round. Diastolic values remained relatively consistent within and between sessions and devices. Overall, values were in the range of normotension.

**Table 3:** Descriptive statistics of blood pressure measurements (mean ± SD).

Measurement type	Iftar (mean ± SD)	Sahoor (mean ± SD)
1st electronic—systolic	116.29 ± 9.48 mmHg	117.34 ± 9.69 mmHg
1st electronic—diastolic	74.06 ± 6.86 mmHg	75.13 ± 6.32 mmHg
1st watch—systolic	116.38 ± 14.26 mmHg	116.83 ± 9.54 mmHg
1st watch—diastolic	74.32 ± 7.06 mmHg	73.37 ± 7.42 mmHg
2nd electronic—systolic	121.84 ± 11.06 mmHg	121.61 ± 9.60 mmHg
2nd electronic—diastolic	77.21 ± 8.01 mmHg	77.48 ± 7.06 mmHg
2nd watch—systolic	121.31 ± 9.45 mmHg	121.42 ± 8.61 mmHg
2nd watch—diastolic	77.18 ± 7.67 mmHg	76.22 ± 7.04 mmHg

Mean ± standard deviation of systolic and diastolic blood pressure (mmHg) recorded by electronic and smartwatch devices during Iftar and Sahoor, based on first and second readings. SYS, systolic blood pressure; DYS, diastolic blood pressure. Measurements were collected under controlled conditions before (Sahoor) and after (Iftar) fasting.

### 3.2 Time-of-day effects: Iftar vs. Sahoor

A paired t-test was performed to compare the fasting phase (Sahoor vs. Iftar) fasting phase differences in BP. The systolic readings recorded by the smartwatch were comparable between the Iftar and Sahoor in both measurement rounds, as illustrated in Table 4 (*p* > 0.05). However, there were significant differences found for the first and second diastolic readings (*p* < 0.001), with higher readings generally recorded at Iftar. 12 These observations indicate a stronger time-of-day effect in diastolic than systolic BP.

**Table 4:** Paired *t*-test results comparing blood pressure between fasting phases (sahoor vs. Iftar) Using Smartwatch Readings.

Measurement	*t*-statistic	*p*-value	Significant (*p* < 0.05)
1st watch—systolic	−1.54	0.124	No
1st watch—diastolic	5.51	3.84 × 10^−^⁸	Yes
2nd watch—systolic	−0.51	0.613	No
2nd watch—diastolic	5.81	7.00 × 10^−^⁹	Yes

Comparisons reflect within-subject changes between Sahoor (pre-fast) and Iftar (post-fast) timepoints.

### 3.3 Device comparison: electronic vs. watch

In order to evaluate the agreement between devices, at each time point we analysed the reading recorded by the electronic monitor (as the reference) with that of the smartwatch. Paired t-test results are presented in Table 3. There were dramatic disparities in several comparisons. There were significant differences between devices in the second systolic reading at Iftar, and both diastolic readings at Saḥūr (*p* < 0.001). This indicates potential measurement bias or physiological variability not captured equivalently by both tools. As shown in Table 5, although mean measurement error was small across fasting states, the wide limits of agreement—particularly for systolic blood pressure—indicate substantial variability between wearable and reference measurements. Table 6. Illustrates paired t-Test results comparing electronic and smartwatch devices at each timepoint.

**Table 5:** Measurement error summary (watch−electronic).

Parameter	Mean error (mmHg)	SD (mmHg)	95% limits of agreement (mmHg)
SBP—Sahoor	+0.9	9.8	−18.3 to +20.1
SBP—Iftar	+1.4	10.2	−18.6 to +21.4
DBP—Sahoor	−0.6	6.1	−12.6 to +11.4
DBP—Iftar	−0.2	6.4	−12.7 to +12.3

Measurement error was defined as wearable device−electronic monitor. Limits of agreement were calculated as mean error ± 1.96 × SD.

**Table 6:** Paired *t*-test results comparing electronic and smartwatch devices at each timepoint.

Measurement	*t*-Statistic	*p*-Value	Significant (*p* < 0.05)
1st systolic (Iftar)	−0.38	0.708	No
1st diastolic (Iftar)	−2.45	0.014	Yes
2nd systolic (Iftar)	3.45	5.6 × 10^−^⁴	Yes
2nd diastolic (Iftar)	0.37	0.709	No
1st systolic (Sahoor)	3.90	1.0 × 10^−^⁴	Yes
1st diastolic (Sahoor)	18.38	< 0.0001	Yes
2nd systolic (Sahoor)	1.43	0.154	No
2nd diastolic (Sahoor)	13.74	< 0.0001	Yes

Paired comparisons evaluate the consistency and agreement between the Huawei Watch D and electronic upper-arm BP monitor.

## 4 Correlation between devices

All conditions saw smartwatch measurements calculated which appear in Table 7. Analysis revealed complex and substantial correlations for both systolic and diastolic measurements across all periods (r = 0.63–0.75 *p* < 0.001) with diastolic readings showing peak values during Iftar (r = 0.754) and Sahoor (r = 0.711). The data suggests the two instruments show linear agreement particularly for diastolic pressure.

**Table 7:** Pearson correlation coefficients between electronic and smartwatch blood pressure readings.

Time	Round	Measurement type	Pearson r	*p*-value	Interpretation
Iftar	1st	Systolic (SBP)	0.390	1.3 × 10^−^¹²³	Moderate correlation
Iftar	1st	Diastolic (DBP)	0.590	< 0.0001	Strong correlation
Iftar	2nd	Systolic (SBP)	0.630	< 0.0001	Strong correlation
Iftar	2nd	Diastolic (DBP)	0.754	< 0.0001	Very strong correlation
Sahoor	1st	Systolic (SBP)	0.683	< 0.0001	Strong correlation
Sahoor	1st	Diastolic (DBP)	0.681	< 0.0001	Strong correlation
Sahoor	2nd	Systolic (SBP)	0.625	< 0.0001	Strong correlation
Sahoor	2nd	Diastolic (DBP)	0.711	< 0.0001	Very strong correlation

Pearson correlation coefficients reflect the linear association between systolic and diastolic blood pressure measurements from the Huawei Watch D and the electronic reference monitor across both fasting (Sahoor) and rehydration (Iftar) periods.

**Table 8:** Regression model performance: predicting electronic from watch data.

Metric	Systolic (SBP)	Diastolic (DBP)
Mean squared error (MSE)	47.43 mmHg²	16.53 mmHg²
R² score (explained var)	0.45	0.60

Performance metrics for linear regression models using watch data to predict electronic blood pressure readings.

**Figure 1:**
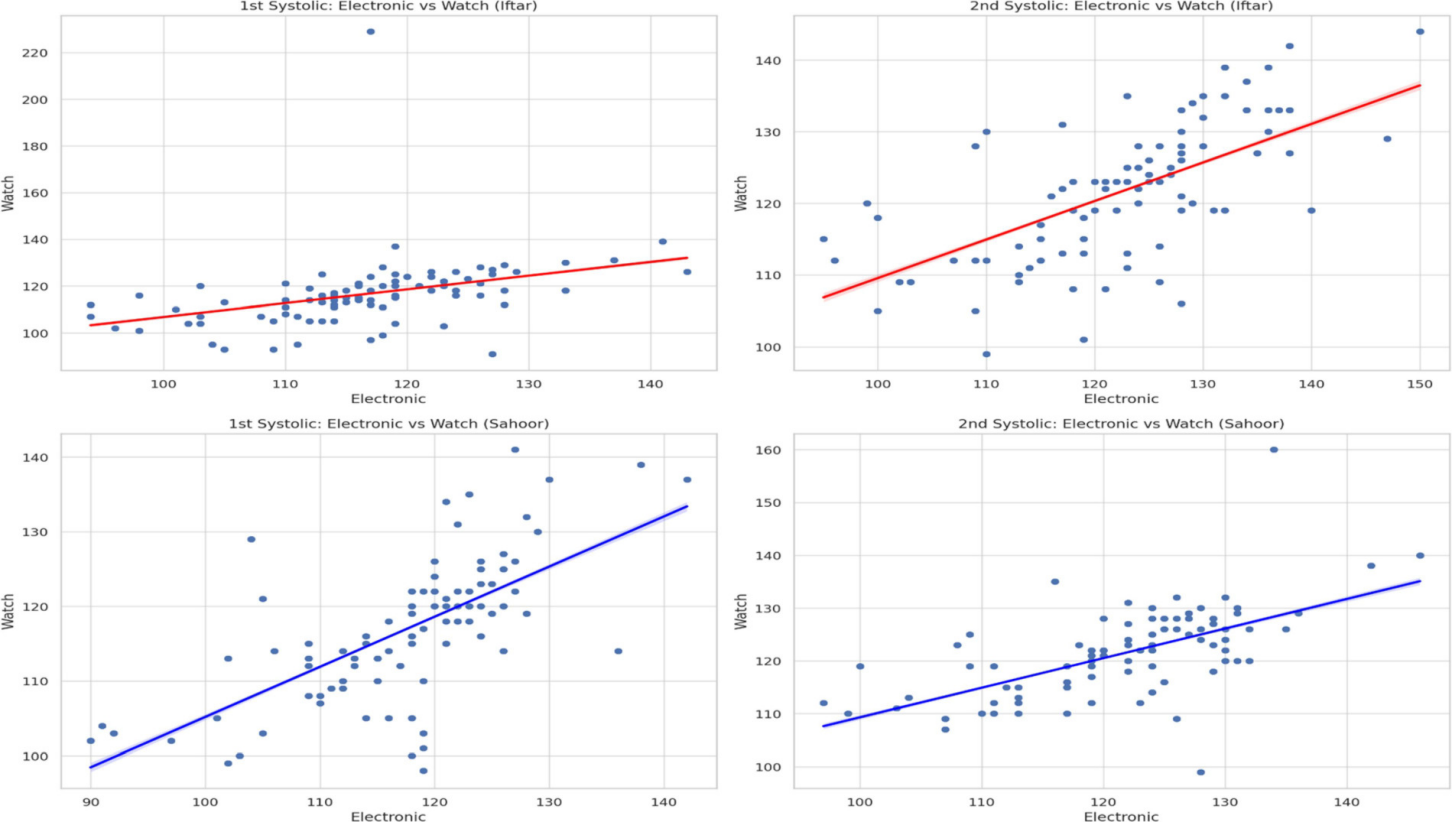
Electronic vs. watch systolic blood pressure measurements—Iftar. Comparison of systolic measurements between devices at Iftar time.

**Figure 2:**
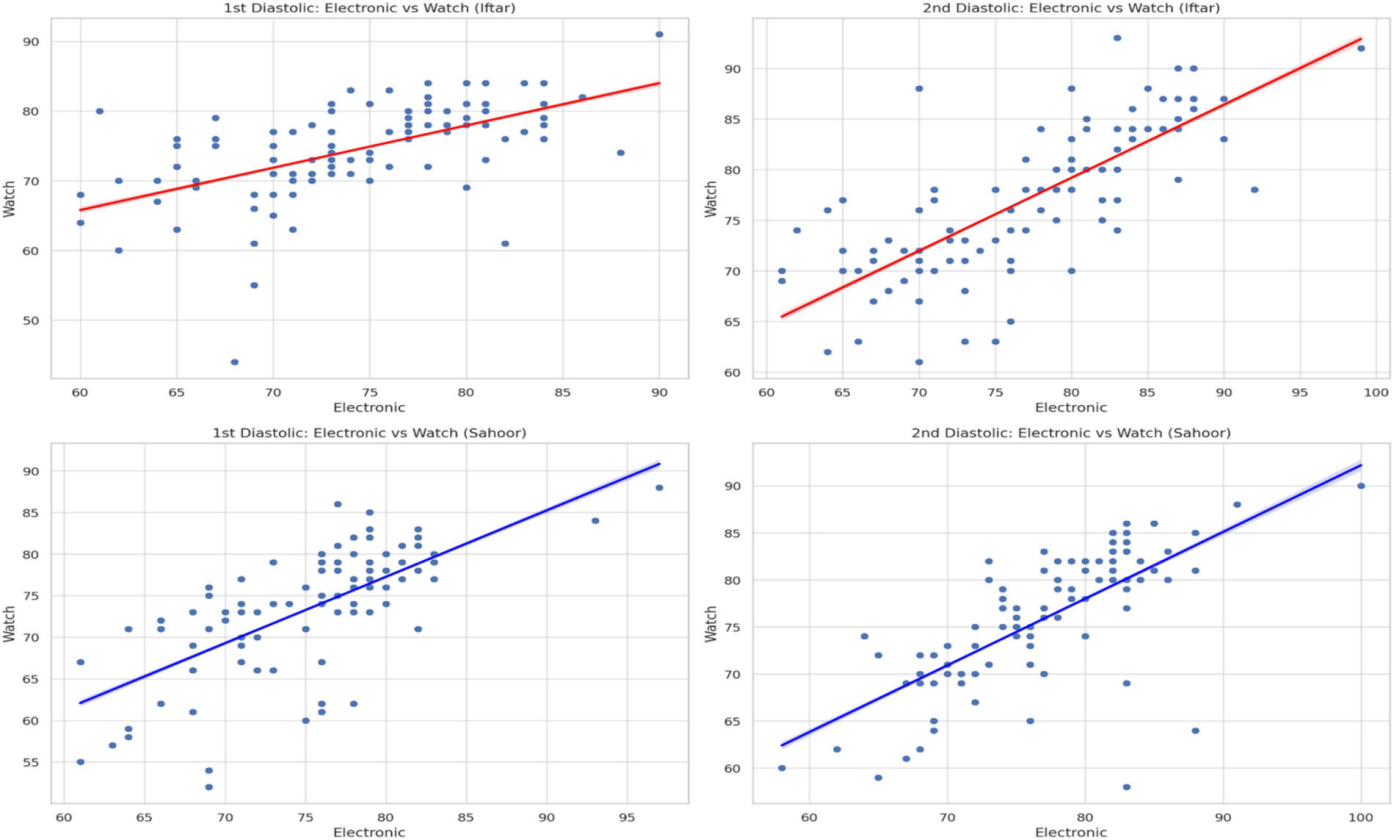
Electronic vs. watch diastolic blood pressure measurements—Iftar. Comparison of diastolic measurements between devices at Sahoor time.

Figures 1, 2 visually support these findings by showing scatter plots of electronic vs. watch readings, with a clear positive linear trend across both systolic and diastolic values. Notably, the clustering of points around the regression line is tighter for diastolic values, which is consistent with the higher correlation coefficients.

## 5 Bland-Altman analysis

**Figure 3:**
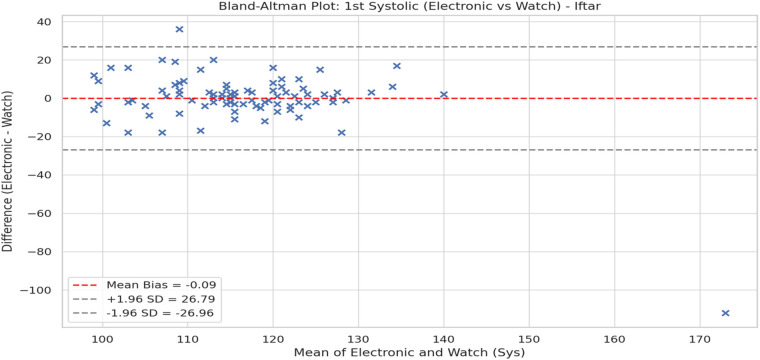
Bland-Altman plot: 1st systolic (electronic vs. watch)—Iftar agreement analysis showing bias and limits of agreement.

**Figure 4:**
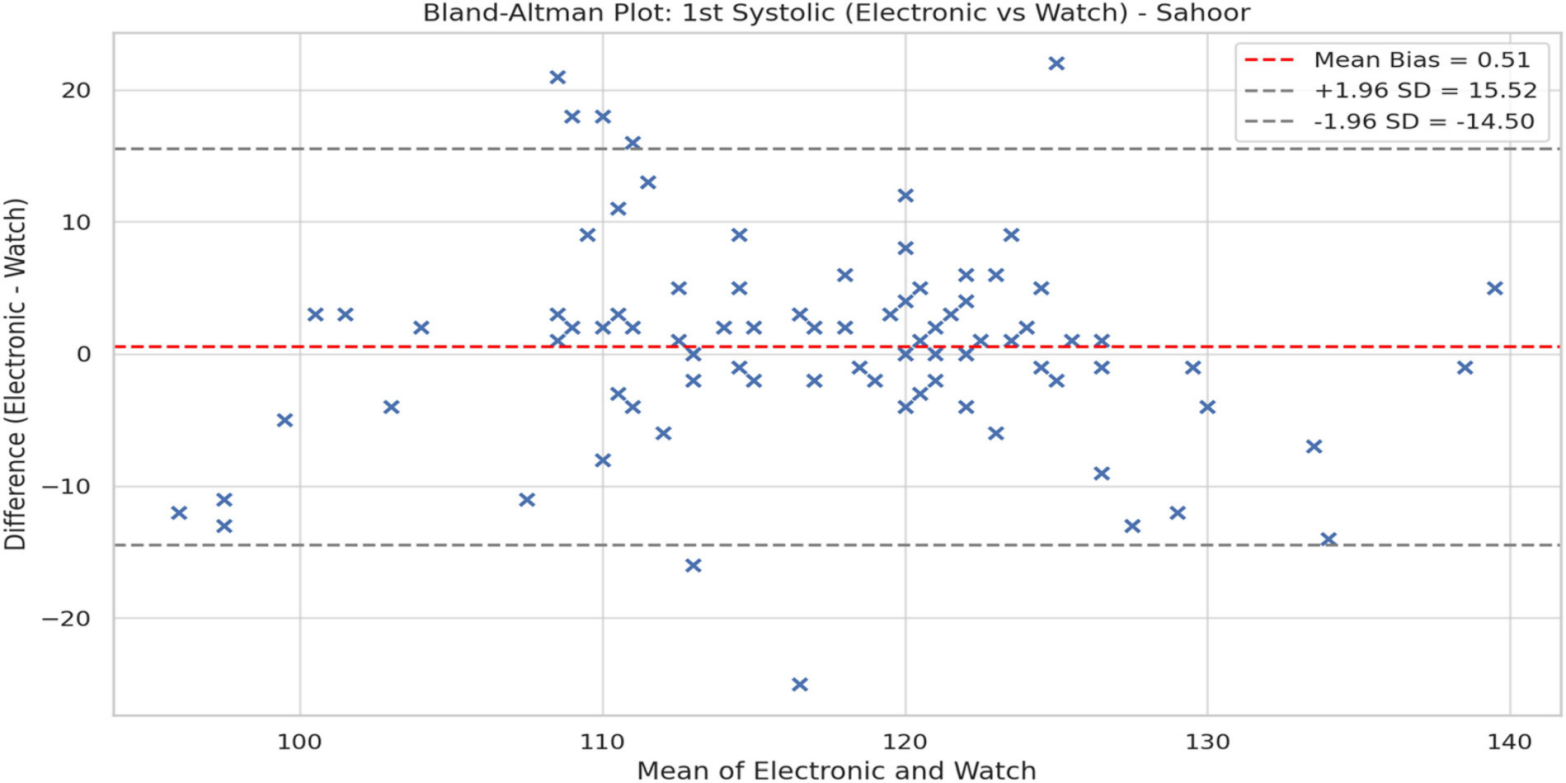
Bland-Altman plot: 1st systolic (electronic vs. watch)—Sahoor Bland-Altman plot for systolic comparison during Sahoor.

The two devices were also compared under Bland-Altman plots. Figure 3 is the Bland-Altman plot for the initial systolic reading at Iftar and Figure 4 is the Bland-Altman plot for Sahoor. Both plots show small average bias (±1 mmHg) with no indication of significant fixed error. Nevertheless, the broad LoAs (about ±20 mmHg) indicate significant interindividual variation and potential limitations in interchangeability between the 2 devices.

## 6 Measurement error and agreement analysis

The primary evaluation metric in this study was measurement error, defined as the difference between blood pressure values obtained from the Huawei Watch D and the electronic upper-arm monitor (Watch−Electronic). Measurement error was analyzed separately for SBP and DBP and stratified by fasting state (Sahoor vs. Iftar).

For systolic BP, the mean measurement error across participants was small (approximately ±1 mmHg), indicating minimal systematic bias between devices. However, the standard deviation of the error was relatively large, resulting in wide 95% limits of agreement approaching ±20 mmHg, as confirmed by Bland–Altman analysis. This reflects substantial inter-individual variability despite the low average bias.

In contrast, diastolic BP exhibited smaller error dispersion, with narrower limits of agreement and lower variability across both Sahoor and Iftar measurements. Error distributions for DBP were more tightly clustered around zero, indicating better consistency between the wearable and electronic monitor under both fasting and rehydration conditions.

When stratified by fasting phase, no meaningful shift in mean error was observed between Sahoor and Iftar for either SBP or DBP. However, variability remained consistently higher for systolic measurements in both phases, whereas diastolic measurements demonstrated more stable agreement across physiological states.

## 7 Predictive modeling using wearable data

To assess the potential of smartwatch readings to predict electronic monitor values, linear regression models were trained using wearable systolic and diastolic data as inputs. Performance metrics are summarized in Tables 6, 8. The model predicted diastolic pressure with moderate accuracy (R² = 0.60), while systolic predictions were less accurate (R² = 0.45). This suggests wearable devices may be more reliable for estimating diastolic than systolic blood pressure.

## 7 Clustering of participants by blood pressure profiles

**Figure 5:**
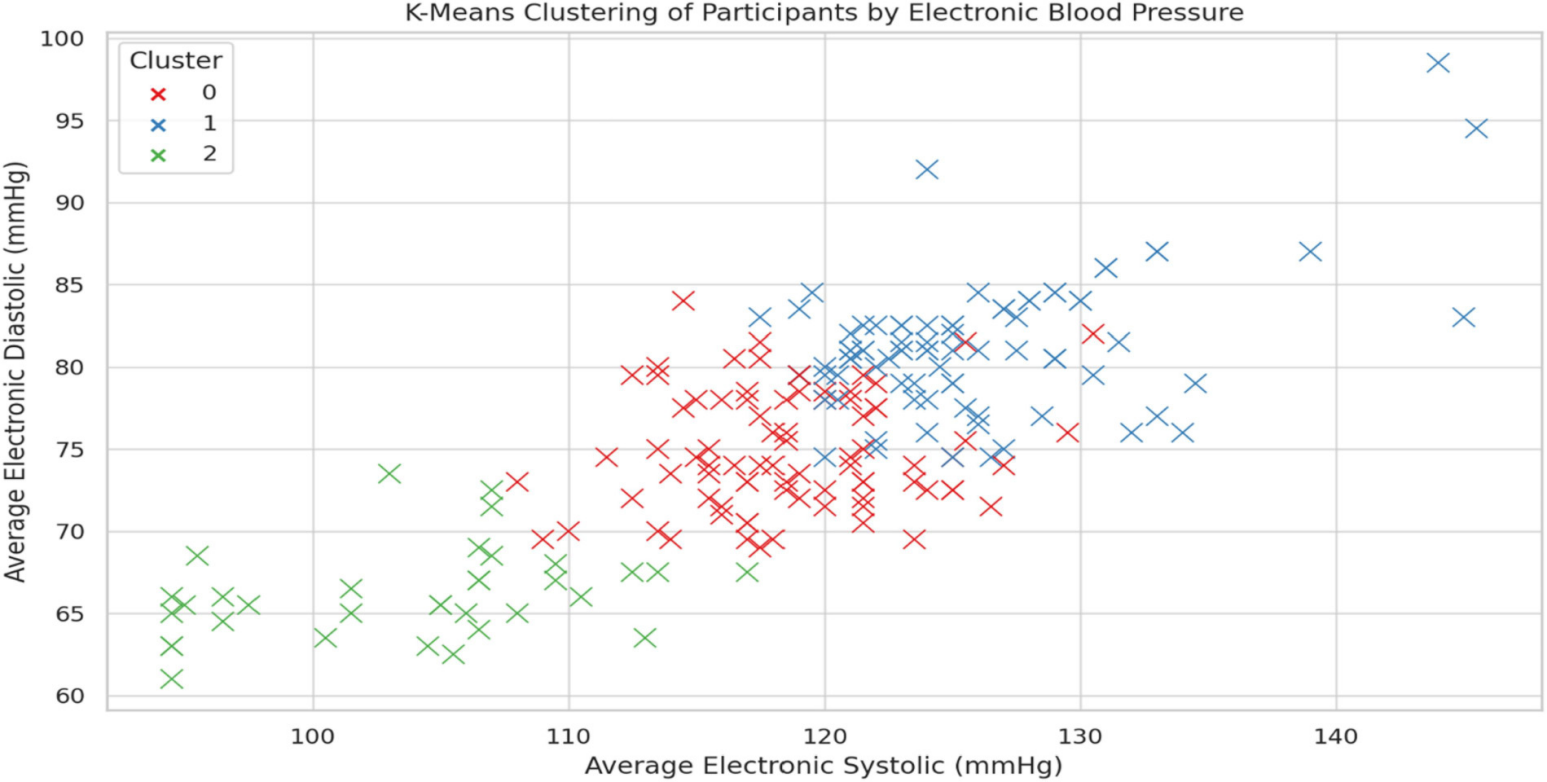
K-Means clustering of participants by electronic blood pressure. Clustered participant groups based on electronic BP readings.

Statistical clustering using k-means was used to characterize subgroups of mobile health users based on their mean electronic blood pressures. In this section, results of the PCA (Figure 5) are discussed in terms of three separate patterns, consisting of normotensive, pre-hypertensive and hypertensive, were observed for cases in the present study. This unmonitored analysis demonstrates the promise of wearable-integrated monitoring for personalized health feedback and at-risk population screening in fasted states.

### 7.1 Comparison of linear and non-linear machine learning models

Non-linear machine learning models demonstrated improved predictive performance compared with linear regression across both systolic and diastolic blood pressure estimation tasks. In particular, Gradient Boosting Regression and SVR (RBF kernel) achieved lower MAE and RMSE values and higher R² scores, indicating superior modeling of the complex relationship between wearable-derived measurements and reference blood pressure values.

Performance gains were more pronounced for systolic blood pressure, which exhibited greater inter-individual variability. Elastic Net regression provided moderate improvement over ordinary linear regression, suggesting that regularization partially mitigates multicollinearity but does not fully capture non-linear physiological effects.

As shown in Table 9, non-linear models—particularly Gradient Boosting and SVR—achieved lower prediction errors and higher explained variance compared with linear regression, supporting the presence of non-linear relationships between wearable and reference blood pressure measurements.

**Table 9:** Comparison of ML model performance.

Model	MAE (mmHg)	RMSE (mmHg)	R²
Linear regression	7.9	10.6	0.48
SVR (RBF)	6.4	8.9	0.63
Gradient boosting	5.8	8.1	0.69
Elastic net	7.1	9.8	0.55

Performance metrics represent averages across systolic and diastolic BP prediction tasks using participant-level cross-validation.

As shown in Tables 10, 11, non-linear models—particularly Gradient Boosting and SVR with an RBF kernel—demonstrated lower prediction errors and higher explained variance than linear regression, with more pronounced improvements for diastolic blood pressure.

**Table 10:** Comparison of machine learning model performance for predicting electronic blood pressure.

Model	MAE (SBP,mmHg)	RMSE (SBP,mmHg)	R² (SBP)	MAE (DBP,mmHg)	RMSE (DBP,mmHg)	R² (DBP)
Linear regression	8.9	11.8	0.42	6.1	8.3	0.55
SVR (RBF kernel)	7.1	9.6	0.58	4.8	6.7	0.69
Gradient boosting	6.5	8.9	0.64	4.3	6.1	0.74
Elastic net (L1 + L2)	7.9	10.7	0.50	5.4	7.6	0.61

Performance metrics were obtained using participant-level cross-validation. Lower MAE/RMSE and higher R² indicate better model performance.

**Table 11:** Comparison of previous studies and the present study on BP monitoring during ramadan.

Aspect	Previous studies	Present study
Objective	Assess BP changes during Ramadan fasting (3, 4)	Evaluate smartwatch accuracy during Sahoor and Iftar; assess wearables for reliable BP estimation
Fasting timing consideration	General Ramadan period; not time-specific (7)	Time-specific comparison between Sahoor (pre-fast) and Iftar (post-fast)
Measurement method	Single-device (oscillometric) readings, often self-reported or clinic-based (4)	Dual-device comparison (oscillometric vs. PPG-based wearable); within-subject repeated measurements
Device type	Mostly upper-arm clinical monitors (3)	Huawei Watch D (wrist-based oscillometric wearable) vs. electronic upper-arm FDA-validated device
Validation of devices	Clinical monitors assumed validated	Both devices validated: Watch D (FDA approved); upper-arm device (ISO 81,060-2 standard)
Use of machine learning	Rarely applied	Applied supervised regression and unsupervised clustering to enhance predictive and subgroup insights
Sample size & design	Smaller or cross-sectional samples (7)	101 participants; within-subject, repeated-measures design
Contextual sensitivity	Limited integration of fasting-specific context	Designed specifically for Ramadan fasting phases to evaluate physiological impact and device usability
Data granularity	Single-time readings or average over month	Multiple readings per participant (Sahoor and Iftar) across multiple sessions

## 8 Discussion

The comparison between the Huawex Watch D smartwatch and the clinically validated upper—arm electronic monitor revealed important insights into the reliability of wearable BP monitoring during fasting and rehydration phases of Ramadan. While both devices followed similar trends, significant differences were observed in several paired measurements. The initial diastolic measurements at Sahoor showed marked variance from subsequent readings and significant discrepancies appeared between second systolic values at Iftar (*p* 0. 001). The findings indicate that wearables offer a seamless and user—friendly non—invasive blood pressure monitoring solution. They exhibit inherent measurement biases under specific physiological conditions particularly in systolic pressure estimation. Prior studies demonstrated that wearable blood pressure monitors using photoplethysmography technology experience performance variability due to factors like hydration levels and vascular tone as well as temperature fluctuations (18, 19).

Comparison of time-of-day differences between Sahoor (pre-fast) and Iftar (post—fast) confirmed a significantly different physiological BP profile, diastolic in particular. Diastolic BP The diastolic BP values were significantly greater post —Iftar in the first two times of measurement with the smartwatch as an indicator of postprandial rise in vascular resistance and volume status. This is against the background of the fact that rehydration following 36 h of fasting increases the circulating blood volume and sympathetic activity induced by diastolic pressure (20, 21). Conversely, systolic BP values remained relatively stable in the different fasting states, indicating that the systolic BP might be less influenced by acute physiologic responses to fasting and rehydration. The results indicate that DBP is an index that is sensitive to changes in cardiovascular state with fasting. Additional correlation analyzes revealed the similarity of devices. Pearson's correlation coefficients revealed strong to very strong linear relationships between the smartwatch and electronic monitor for diastolic BP at both Sahoor and Iftar (r = 0.681–0.754, *p* < 0.001).

Correlations were similarly lower (although still significant; r = 0.390—0. 683) for systolic BP. Such associations were however visually confirmed which suggested better grouping for the diastolic values closer to the regression line in scatterplots. These findings imply that although the smartwatch cannot accurately replace clinical—grade measurements, it can reasonably monitor changes in blood pressure, particularly for DBP. Recent advances in wearable blood pressure monitoring include wristbased oscillometric devices incorporating miniaturized inflatable cuffs, which aim to extend clinically established oscillometric principles into portable and user—friendly form factors suitable for home and ambulatory monitoring (22). The observed stronger agreement for diastolic blood pressure may reflect the performance characteristics of wrist—based oscillometric wearable devices, which remain sensitive to peripheral vascular dynamics during fasting and rehydration states (23).

The findings of the present study are consistent with recent post (2020) literature emphasizing the context—dependent performance of wearable blood pressure devices (4) reported significant inter—individual variability in BP responses during Ramadan, underscoring the importance of time—specific and physiologically contextualized measurements. Similarly, the study highlighted that wearable health technologies are best suited for trend monitoring and early risk identification rather than diagnostic decision—making, particularly under non—standard physiological conditions such as fasting and dehydration (12).

Recent validation—focused reviews (2021—2024) have stressed that wrist—based wearable BP devices, even when employing oscillometric cuff mechanisms, frequently demonstrate wide limits of agreement when compared with upper—arm reference standards. This observation aligns with the present study's measurement error analysis and reinforces the conclusion that wearable BP monitoring should be interpreted as a complementary, rather than substitutive, tool in clinical practice. Wide limits of agreement were identified for both devices, and this was despite the positive associations identified between devices thereby emphasizing intra—individual heterogeneity. The mean bias appeared minimal at approximately 1 mm Hg. However, the differences spanned a broad range reaching ±20 mm Hg. This variability emphasizes intra—individual heterogeneity and indicates that wearable devices, despite their convenience and usability, do not achieve clinical equivalence with standard monitors for individual diagnosis or medication titration. These findings highlight the importance of additional optimization of algorithms and evaluation in various physiological conditions, as those reported in Ramadan fasting. The predominance of young—to—middle aged adults with a mean BMI in the overweight range may partially explain the observed inter—individual variability in systolic blood pressure measurements (24).

The absence of formal AAMI or BHS certification has important implications for the clinical interpretation of wearable blood pressure data. Although ISO 81060-2—aligned validation provides evidence that a device meets internationally recognized accuracy and performance benchmarks under controlled conditions, AAMI and BHS certification represent higher-tier clinical validation frameworks that assess device reliability across broader populations and clinical scenarios (25).

As such, while the Huawei Watch D demonstrates promising agreement with a validated upper-arm oscillometric monitor and offers valuable insight into blood pressure trends during Ramadan fasting, its measurements should be interpreted within a research and screening context rather than for diagnostic decision—making or medication titration. Nevertheless, ISO—aligned validation remains highly relevant for wearable devices, as it supports methodological rigor and enables meaningful comparison with established oscillometric systems in exploratory and population—based studies.

Machine learning incorporated provided additional value to interpreting wearable BP data. Models to predict diastolic BP were moderately accurate using smartwatch readings as predictors (R² = 0.60); predictions for systolic BP were less accurate (R² = 0.45). These findings add to the evidence that diastolic blood pressure obtained from wearables is likely to be a more reliable, clinically useful number than readings of systolic blood pressure, ‘ the researchers wrote. Additionally, unsupervised clustering by K—means resulted in three discrete participant subgroups can be considered as falling into (approximately) normotensive, pre—hypertensive, and hypertensive categories. This unsupervised discovery of risk groups demonstrated the feasibility of wearables to facilitate the risk stratification and early warning systems in community health (26).

Former studies assessing the effect of Ramadan fasting on BP have yielded variable and inconclusive results. BP has been reduced during Ramadan in some studies, possibly because of weight loss and improved metabolic regulation (27, 28). On the other hand, some studies have noted that fasting has been associated with increased BP or BP variability, including dehydration, sleep disturbances, or differences in autonomic activity (29, 30). One of the greatest limitations of these initial studies. However, in methodology many were based on single or average BP measurements collected at one point during the month and (in many cases) in a clinic setting or through self-reports, with no information on whether readings were taken while fasting (Sahoor) or rehydrating (Iftar). Furthermore, these studies applied mostly the standard of an upper-arm oscillometric monitor, which, although clinically validated, is not meant to be used as the primary tool for frequent or real-life observation in natural settings. Although the Huawei Watch D employs an oscillometric cuff-based approach, wrist-based measurements remain more susceptible to positioning and vascular variability compared to upper-arm devices, which may partially explain the observed limits of agreement. A further limitation of this study is that the Huawei Watch D, while validated in accordance with ISO 81060-2–aligned protocols, lacks formal AAMI or BHS certification. This limits the generalizability of the findings to diagnostic clinical use and reinforces that the results should be interpreted primarily in the context of trend monitoring and research applications.

In contrast, the present study introduces several methodological advancements. It adopts a within-subject, repeated-measures design with 101 participants, enabling comparison of BP readings at two distinct physiological states: pre-fast (Sahoor) and post-fast (Iftar). It also incorporates both a clinically validated electronic monitor and a wearable smartwatch (Huawei Watch D), allowing for a head-to-head comparison between oscillometric and PPG-based BP estimation. These two machines have received FDA approval or validation in accordance with ISO 81060-2 protocols, attesting to their high accuracy. In addition to robust statistical analysis such as paired t tests, Bland–Altman plots, and Pearson correlation, this study uses machine learning models for regression and unsupervised clustering. This mixed-methods approach brings richness to our understanding of the performance of technology, individual differences, and the potential of wearables in “culturally sensitive” health domains, which was lacking in previous studies.

Remarkable and unexpected was the fortuitous discovery of hyper-BP in subjects who reported they were healthy and who followed no antihypertensive therapy. Notwithstanding exclusion criteria to select for normotensives, cluster analysis demonstrated that many had mean BP compatible with pre-hypertension or hypertension, especially at Sahoor, when there is a greater degree of physiologic stress and dehydration. This finding demonstrates wearable devices’ potential to not only be used as a reference monitor of established conditions, but also as a tool to screen for undiagnosed hypertension—a particularly valuable addition to preventive healthcare in regions with limited access to routine screening. The superior performance of non-linear machine learning models supports the hypothesis that the relationship between wearable blood pressure measurements and reference values is not strictly linear, particularly under physiological perturbations such as fasting and postprandial states. Blood pressure regulation is governed by complex, non-linear interactions involving vascular compliance, autonomic nervous system activity, hydration status, and circadian rhythms.

Gradient boosting and kernel-based models are better suited to capture these nonlinear dependencies, which likely explains their improved predictive accuracy compared with linear regression. These findings reinforce recent recommendations that wearable blood pressure analytics should incorporate non-linear modeling approaches, particularly when applied to dynamic physiological contexts such as Ramadan fasting.

This study overall underlines the value and restrictions of the wearable BP monitor technology during Ramadan. The investigation emphasizes both the potential benefits and limitations of wearable blood pressure monitoring technology during Ramadan. Our device provides essential physiological feedback during fasting periods, which routine medical care often neglects, even though it cannot replace clinical lab tests. The findings of the research indicate that wearable technology can contribute to improving personalized and culturally sensitive health monitoring for Muslims fasting during Ramadan due to its capability to provide context aware real—time feedback. The inherent biases, together with apparent inconsistencies, prompt extensive improvement and validation before these assays can be broadly used in diagnostics.

From a clinical perspective, the observed measurement error distribution has important implications for the practical use of wearable blood pressure devices. Although the mean bias between the Huawei Watch D and the electronic upper—arm monitor was small, the wide limits of agreement (±20 mmHg) — particularly for systolic blood pressure—indicate that the two devices are not diagnostically interchangeable at the individual level. Such variability exceeds clinically acceptable thresholds for hypertension diagnosis and medication titration, where precise absolute values are required (31).

Nevertheless, the relatively narrower error distribution observed for diastolic blood pressure suggests that wrist based oscillometric wearables may be suitable for longitudinal trend monitoring and population-level screening, especially in settings where conventional measurements are impractical. In the context of Ramadan fasting, where hydration status and daily routines vary substantially, wearable devices may provide valuable insight into directional BP changes and temporal patterns, even if they cannot replace clinical—grade measurements for diagnostic decision—making. The improved performance of non— linear models supports the presence of non—linear physiological relationships between wearable and reference blood pressure measurements, especially for systolic pressure under fasting—related variability. The observed limits of agreement of approximately ±20 mmHg for systolic blood pressure suggest that the wearable device is suitable for trend monitoring and screening, but not for diagnostic decision—making or antihypertensive treatment adjustment.

Accordingly, wearable BP monitoring should be viewed as a complementary tool for early risk identification, behavioral monitoring, and research applications, rather than a substitute for validated clinical sphygmomanometry. This investigation has direct relevance to contemporary healthcare, especially as it pertains to the development of remote, culturally enabled cardiovascular monitoring. The accuracy of the diastolic blood pressure detection effect of wearable devices such as the Huawei Watch D under fasting conditions indicates their potential use in ongoing real world health monitoring. Diastolic BP variations noticed between fasting and rehydration time periods, in particular, a higher value post—Iftar, are in concordance with other literature, which states that such hemodynamic reactions as renascent sympathetic tone and plasma volume increase after fluid and energy intake also exist. In populations from Ramadan—observing countries or among individuals who fast, traditional clinic BP measurement is unrealistic because of changes in daily routine, dehydration, and inadequate access to the clinic.

In addition, the incidental detection of high blood pressure in self—reported healthy individuals highlights the potential application of wearables in early detection of underdiagnosed hypertension, which is crucial for preventative healthcare. This is in line with the increasing evidence on the utility of wearable health tech for early detection of cardiovascular risk factors outside healthcare settings (32, 33). The machine learning approaches used— namely user clustering into normotensive, pre—hypertensive, and hypertensive—also demonstrate the scalability of such tools for population risk stratification systems and the value of these models in public health applications. The study also bridges a critical gap by addressing device performance under non-standard physiological states, where hydration, peripheral vascular tone, and autonomic regulation vary dramatically—conditions under which most wearables are rarely tested. While systolic readings from wearables remain less reliable and require further algorithmic refinement, the observed agreement in diastolic measurements supports a cautious but meaningful integration of wearable BP monitoring into hypertension screening programs, medication adherence strategies, and culturally tailored healthcare during Ramadan and beyond.

## Conclusion

This paper assessed the performance of a wrist—worn blood pressure monitoring device (the Huawei Watch D) under the different physiological fasting (Sahoor) and rehydration (Iftar) states in Ramadan compared to a validated upper—arm monitor. Significant correlations were found, especially for diastolic pressure, which indicated that wearable devices were valid for monitoring overall trends in BP under fasting status. But significant differences and broad limits of agreement between devices. Particularly for systolic readings, mean could be right to rely on wearable—based readings as a substitute for those taken by clinicians for diagnosing ills. The study confirmed diastolic blood pressure as an earlier index of fast—related physiological changes. They showed the possibility of detecting at risk individuals by using data from wearables in data driven methods with clustering training even if individuals are unaware of their elevated BP. Also, They showed that wearable data could be used as a support of hypertension screening in out of—office setting, where fast need to be detected. These results indicate that with a able contextual interpretation, wearable BP can contribute to increasing health awareness and provide early risk detection, as well as culturally oriented monitoring during Ramadan. However, additional studies and regulatory validation are required to improve wearable algorithms and to establish their reliability in heterogeneous populations and clinical conditions.

## Data Availability

The datasets presented in this study can be found in online repositories. The names of the repository/repositories and accession number(s) can be found below: https://ieee-dataport.org/documents/blood-pressure.
